# Co-Delivery of a Novel Lipidated TLR7/8 Agonist and Hemagglutinin-Based Influenza Antigen Using Silica Nanoparticles Promotes Enhanced Immune Responses

**DOI:** 10.3390/pharmaceutics16010107

**Published:** 2024-01-13

**Authors:** Walid M. Abdelwahab, Sarah Auclair, Timothy Borgogna, Karthik Siram, Alexander Riffey, Hélène G. Bazin, Howard B. Cottam, Tomoko Hayashi, Jay T. Evans, David J. Burkhart

**Affiliations:** 1 Center for Translational Medicine, University of Montana, Missoula, MT 59812, USAkarthik.siram@mso.umt.edu (K.S.); alexander.riffey@mso.umt.edu (A.R.); jay.evans@mso.umt.edu (J.T.E.); 2 Department of Biomedical and Pharmaceutical Sciences, University of Montana, Missoula, MT 59812, USA; 3 Inimmune Corporation, 1121 East Broadway, Missoula, MT 59812, USA; helene.g.bazinlee@inimmune.com; 4 Moores Cancer Center, University of California San Diego, La Jolla, CA 92093, USAthayashi@health.ucsd.edu (T.H.)

**Keywords:** co-delivery, silica nanoparticles, vaccine adjuvant, toll-like receptor 7/8 (TLR7/8) ligand, INI-4001, influenza virus, hemagglutinin H7

## Abstract

Co-delivery of antigens and adjuvants to the same antigen-presenting cells (APCs) can significantly improve the efficacy and safety profiles of vaccines. Here, we report amine-grafted silica nanoparticles (A-SNP) as a tunable vaccine co-delivery platform for TLR7/8 agonists along with the recombinant influenza antigen hemagglutinin H7 (H7) to APCs. A-SNP of two different sizes (50 and 200 nm) were prepared and coated with INI-4001 at different coating densities, followed by co-adsorption of H7. Both INI-4001 and H7 showed >90% adsorption to the tested A-SNP formulations. TNF-α and IFN-α cytokine release by human peripheral blood mononuclear cells as well as TNF-α, IL-6, and IL-12 release by mouse bone marrow-derived dendritic cells revealed that the potency of the INI-4001-adsorbed A-SNP (INI-4001/A-SNP) formulations was improved relative to aqueous formulation control. This improved potency was dependent on particle size and ligand coating density. In addition, slow-release profiles of INI-4001 were measured from INI-4001/A-SNP formulations in plasma with 30–50% INI-4001 released after 7 days. In vivo murine immunization studies demonstrated significantly improved H7-specific humoral and Th1/Th17-polarized T cell immune responses with no observed adverse reactions. Low-density 50 nm INI-4001/A-SNP elicited significantly higher IFN-γ and IL-17 induction over that of the H7 antigen-only group and INI-4001 aqueous formulation controls. In summary, this work introduces an effective and biocompatible SNP-based co-delivery platform that enhances the immunogenicity of TLR7/8 agonist-adjuvanted subunit influenza vaccines.

## 1. Introduction

Vaccines are a highly effective means of managing existing and emerging infectious diseases [1]. However, enhancing the potency, quality, durability, and safety of responses to vaccines remains a challenge. The pharmacokinetics and presentation of vaccine components to the targeted receptors and immune cells must be carefully considered for the successful orchestration of a desirable immune response [2]. In recent years, nanoparticles have gained much interest in the generation of new vaccines. This is attributed to their ability to protect antigen targets from premature proteolytic degradation, resulting in enhanced uptake of antigens by immune cells and facilitating their processing and presentation [3,4]. Nanoparticles can also help create beneficial local inflammation, target lymph node delivery, and alter the kinetics of vaccine delivery [2].

Current licensed subunit vaccines comprised of recombinant protein antigens exhibit favorable safety and immunogenicity profiles. However, pairing these antigens with appropriate adjuvant(s) and a suitable delivery system is imperative to eliciting safe and effective humoral and cellular immune responses [5]. Adjuvants in particular can potentiate immune responses to vaccine antigens, aiming to maximize protective immunity. This becomes of critical importance in higher-risk populations such as the elderly, newborns, and immunocompromised individuals [6]. Most licensed seasonal influenza vaccines are non-adjuvanted and rely primarily on vaccine-induced antibody titers for protection [7]. It is estimated that between 294,000 and 518,000 people (globally) die every year of influenza virus infections and associated complications [8]. Current vaccines are mainly focused on eliciting a strain-matched humoral immune response, requiring a yearly booster, and do not provide supraseasonal protection due to waning immunity and antigenic drift [9]. Adjuvants have been recognized as a key component of influenza vaccines and a major strategy to improving vaccinations and conferring a robust immune response induced by influenza immunogens [8,10]. Although the existing adjuvanted influenza vaccines elicit strong antigen-specific antibody responses, they fail to provide effective, long-term protection—partly due to the absence of robust cellular immunity [8]. To overcome the shortcomings of current influenza vaccines, novel adjuvants and delivery systems that elicit robust and durable humoral and cellular immunity and are capable of providing effective supraseasonal protection are urgently needed [9].

Adjuvants that target specific innate immune receptors are powerful weapons for combating infectious diseases [11,12]. These adjuvants carry pathogen-associated molecular patterns (PAMPs), which trigger pattern recognition receptors (PRRs) including Toll-like receptors (TLRs) and cytosolic immune receptors [8]. TLRs are the best characterized family of PRR-targeting adjuvants. Activation of TLR7/8 boosts antigen presentation by DCs and macrophages as part of the multifaceted adaptive immune response [13]. The broad expression profiles of TLR7/8, poor pharmacokinetic properties of some TLR7/8 ligands, and toxicities associated with systemic administration are barriers to successful clinical translation [14]. Control over the pharmacokinetics, pharmacodynamics, and presentation of these compounds is crucial for their translation into the clinic, emphasizing the need for optimized drug delivery approaches [14].

**Figure 1:**
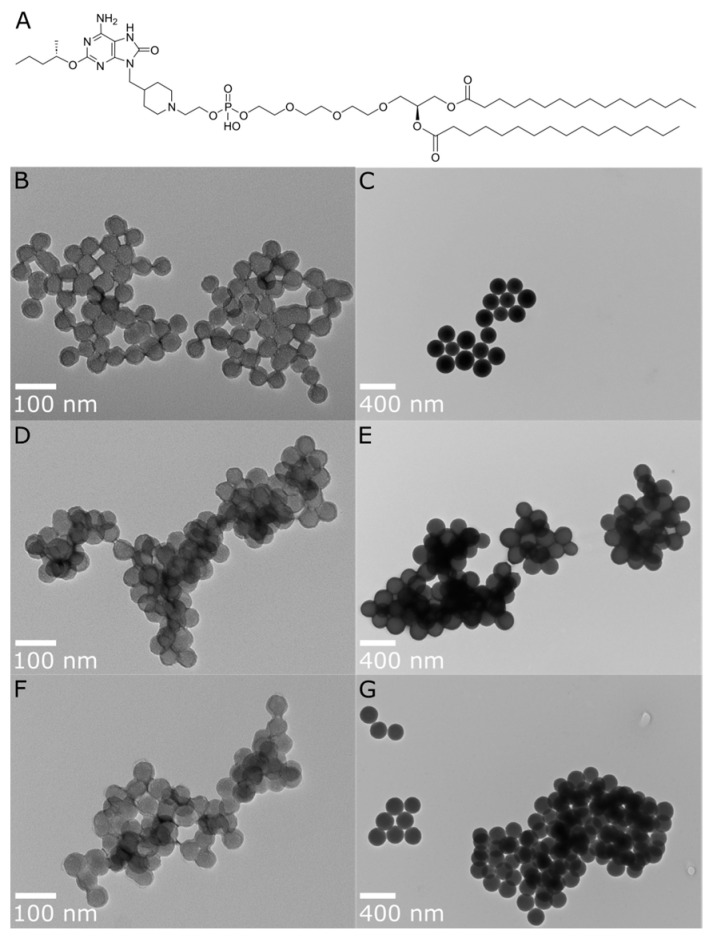
(**A**) Chemical structure of INI-4001 and TEM images for post-functionalization A-SNP of different sizes, with and without adsorbed INI-4001: (**B**) A-SNP-50, (**C**) A-SNP-200, (**D**) INI-4001/A-SNP-50 (low adjuvant coating density), (**E**) INI-4001/A-SNP-200 (low adjuvant coating density), (**F**) INI-4001/A-SNP-50 (high adjuvant coating density), (**G**) INI-4001/A-SNP-200 (high adjuvant coating density). Images were taken at 98,000× and 23,000× magnification, with scale bars indicating 100 nm and 400 nm for (**B**,**D**,**F**) and (**C**,**E**,**G**), respectively. Images are representative of each formulation.

Engineered nanoscale particles can improve the immunogenicity of subunit vaccines by presenting the antigens and adjuvants in a spatial/temporal context more readily recognized by the immune system [2]. In addition, multiple approaches to the conjugation of these TLR7/8 compounds onto particles or directly to the antigen have been effective in modulating the pharmacokinetics and pharmacodynamics of these analogues for therapeutic and prophylactic vaccines [14,15,16,17]. In this report, a novel synthetic lipidated oxoadenine TLR7/8 ligand, INI-4001 (Figure 1), was explored for its ability to elicit strong antigen-specific humoral and T cell-mediated responses to the recombinant influenza virus antigen hemagglutinin H7 (H7) in mice. To achieve this goal, both components, INI-4001 and H7, were co-adsorbed and presented on the surface of tunable amine-grafted silica nanoparticles (A-SNP). INI-4001 acts through both human TLR7 and TLR8 receptors and elicits IFN-α as well as pro-inflammatory cytokines from human PBMCs. Further, INI-4001 results in a strong humoral and Th1-biased cellular immune response when used as a vaccine adjuvant in mice [18].

Silica nanoparticles (SNP) are a promising vaccine delivery platform due to their excellent biocompatibility, physicochemical stability, easily controllable morphology, and tunable surface chemistry [19,20]. Furthermore, their synthesis, functionalization, toxicity, biodistribution, and controlled release properties have been extensively investigated [21,22]. In addition, several human clinical trials using different types of SNP have demonstrated the safety of this delivery system [23,24,25]. Factors such as particle size, surface charge, surface functionality, antigen and adjuvant presentation, and co-delivery can be optimized to alter the vaccine-induced immune responses. For example, small-sized nanocarriers (10–100 nm) migrate to and accumulate in lymph nodes, where they are efficiently captured by resident DCs. In contrast, large nanocarriers are inefficient at infiltrating lymph nodes and are more likely to be captured by circulating phagocytes in the periphery [26,27]. Furthermore, the surface charge of nanoparticles can play a role in their interaction with and subsequent activation of DCs and play a critical role in the adsorption of antigens and/or adjuvants, providing a “depot effect” [28].

Herein, A-SNP of two sizes (50 and 200 nm) were used to achieve co-adsorption of the anionic antigen, H7, and adjuvant, INI-4001, at two different coating densities. We hypothesized that prolonged co-delivery and presentation of the influenza antigen and TLR7/8 agonist using A-SNP as tunable nanoparticles, mirroring the influenza virus-related innate signaling pathways, could elicit strong cellular and humoral immunity. Furthermore, adsorption of a lipidated TLR7/8 agonist onto A-SNP should limit systemic distribution of the TLR7/8 agonist and help improve the safety and efficacy profiles of the adjuvated vaccine. Using this simple but efficient co-adsorption strategy eliminates the need for extensive multi-step conjugation and characterization that can hamper scale-up, advancement, and clinical translation of these vaccine formulations.

In this work, we demonstrate that different sizes of A-SNP can efficiently co-adsorb INI-4001 and lead to strong cytokine induction by human peripheral blood mononuclear cells (hPBMCs) and murine bone marrow-derived dendritic cells (mBMDCs). The potency of these INI-4001-adsorbed A-SNP (INI-4001/A-SNP) formulations was dependent on the particle size and adjuvant-coating density. Antibody and cell-mediated immune responses against the influenza virus H7 antigen were enhanced and broadened when presented on the surface of tunable A-SNP and co-delivered with INI-4001. Furthermore, the magnitude and degree of T-cell polarization were highly dependent on the formulation and vaccination schedule used.

Co-delivery of antigen and adjuvant on A-SNP enhances both humoral and cell-mediated immunity and could be beneficial for anti-viral immunity and the advancement of more potent, next-generation vaccines for influenza A and other indications.

## 2. Materials and Methods

### 2.1. Materials

INI-4001 was synthesized and processed to over 99% purity as described previously [18,29]. Hemagglutinin antigen trimer (H7) was obtained from Dr. Florian Krammer at the Icahn School of Medicine at Mount Sinai [30,31]. All chemicals used were of analytical reagent grade and all solvents were of HPLC grade. Trifluoroacetic acid (TFA) and ammonium hydroxide were obtained from J.T. Baker (Phillipsburg, NJ, USA). Sterile water for irrigation (WFI) was obtained from Baxter Healthcare Corp (Deerfield, IL, USA). Methanol, anhydrous ethanol, isopropyl alcohol, Triton X-100, glycerol, ammonium formate, acetone, tetrahydrofuran, methyl t-butyl ether, and sodium hydroxide were obtained from Thermo Fisher Scientific (Waltham, MA, USA). (3-Aminopropyl) triethoxysilane (APTES) was obtained from Sigma-Aldrich (St. Louis, MO, USA). 50 and 200 nm solid SNP were purchased as ethanol suspensions (10 mg/mL) from NanoComposix (San Diego, CA, USA). RPMI medium 1640 (Cat. No. 11875-093, Gibco, Thermo Fisher Scientific, Waltham, MA, USA) was supplemented with 10% fetal bovine serum (Cat. No. 35-011-CV, Corning Inc., Corning, NY, USA), 100 U/mL penicillin, 100 μg/mL streptomycin, and 292 μg/mL glutamine (Cat. No. 10378-016, Thermo Fisher) to prepare the complete RPMI media (RP-10).

### 2.2. Preparation of A-SNP

The surface of the 50 and 200 nm bare SNP was decorated with cationic aminopropyl groups using a post-synthesis grafting sonochemical method [32,33]. Briefly, a suspension containing 500 mg of bare SNP of each size was transferred to a sterile 50 mL centrifuge tube and washed 2 times with WFI and 3 times with ethanol before drying under reduced pressure at 60 °C for 8 h using a vacuum oven. Twenty-five mL suspensions of the dried powders were prepared in WFI at a 10 mg/mL concentration in separate 100 mL round-bottom flasks followed by sonication for 10 min to fully disperse the particles. For surface amine functionalization, the reaction flasks were charged with 25 µL of APTES followed by sonication and frequent stirring for 3 h at 75 °C. The A-SNP were isolated by centrifugation (3500× *g*, 30 min) and washed several times with WFI and ethanol to remove any unreacted APTES. After washing, particles were dried under reduced pressure at 60 °C for 8 h using a vacuum oven.

### 2.3. Co-Adsorption of Adjuvant (INI-4001) and Antigen (H7) onto A-SNP

Different sizes of A-SNP were used to adsorb INI-4001 according to a thin film rehydration method [20]. Briefly, aliquots of 40 mg/mL of A-SNP suspension and 1 mM of INI-4001 stock solution (both in ethanol) along with 0.2 molar equivalents of choline bicarbonate were added into 2 mL sterile Covaris glass vials (Woburn, MA, USA), and after brief mixing, the solvent was removed under reduced pressure using a rotary evaporator to form the thin films. Five hundred µL of 2% glycerol were added to rehydrate the films at the target concentrations. The formulations were then bath sonicated for 60 min followed by 1 min in the Covaris S2 Ultrasonicator (Woburn, MA, USA) at 15–25 °C to ensure full dispersion of the particles and appropriate coating of the agonist on the beads. The blank A-SNP and INI-4001 aqueous controls were prepared similarly. To co-adsorb HA antigen (H7) onto INI-4001/A-SNP, an aliquot from the antigen prepared at 0.2 mg/mL in 2% glycerol was then added to the suspended adjuvant-coated A-SNP followed by end-over-end mixing using a rotator for 2 h at room temperature to allow adsorption of the anionic antigen to the cationic particles, mainly via electrostatic interaction. The targeted concentrations of INI-4001 and H7 in these formulations were 240 µM and 20 µg/mL, respectively.

### 2.4. Characterization of INI-4001/A-SNP Formulations

#### 2.4.1. Size Distribution by DLS and Transmission Electron Microscopy (TEM)

Hydrodynamic particle size and PDI were measured by dynamic light scattering (DLS) using Zetasizer Nano-ZS (Malvern Panalytical, Malvern, UK). Samples were diluted to 1:10 in the native diluent, and three measurements were averaged to determine the hydrodynamic diameter and PDI based on the intensity function. The surface morphology and microstructures were analyzed using TEM (Hitachi H-7100 transmission electron microscope). The electron microscopy was performed at the Multiscale Microscopy Core with technical support from the Oregon Health & Science University (OHSU)/FEI Living Lab and the OHSU Center for Spatial Systems Biomedicine. For TEM analysis, 0.1 mg/mL of nanoparticle solution in WFI was prepared. Five μL of the prepared solution were dropped onto the 400-mesh carbon-supported copper grid and dried. The grid was transferred to a TEM holder and inserted into the microscope. Five to ten images were acquired with a magnification of at least 23,000× operated at 75 kV.

#### 2.4.2. Zeta Potential and pH Measurements

To measure the zeta potential of the INI-4001/A-SNP formulations, DLS (Zetasizer, Malvern, UK) was used with a He–Ne laser (633 nm) at 90° to collect optics at 25 °C. Nanoparticle suspensions of 0.1 mg/mL were prepared in 10 mM NaCl (pH 5.5). Seven hundred µL of each solution were transferred in a folded capillary cuvette and used for sample acquisition. The pH was measured from the samples diluted for the zeta potential measurements using an Accumet AB150 pH meter (Thermo Fisher Scientific) and an InLab Micro probe (Mettler-Toledo, Columbus, OH, USA) after a three-point calibration using pH 4.01, 7.00, and 10.01 standards.

#### 2.4.3. Quantitative Analysis and Determination of Adsorption of INI-4001 and H7 onto A-SNP

INI-4001 and H7 were adsorbed onto A-SNP (50 and 200 nm at low and high densities; Table 1) as described in Section 2.3. To determine the INI-4001 concentration, 30 µL from each sample were transferred to a 0.5 mL microcentrifuge tube after brief vortexing to fully suspend the particles. Fifty µL of 3% Triton X-100 were then added and mixed for 1 min to desorb and extract INI-4001 from the surface of the particles. The particles were isolated by centrifugation at 960× *g* for 4 min and the supernatant was transferred to a suitable HPLC vial. The extraction step was repeated twice, and the supernatants were collected in the same HPLC vial. A Waters Acquity Arc UHPLC chromatograph system (Milford, MA, USA) equipped with a quaternary solvent manager-R, an FTN-R sample manager and injector valve with a 20 μL loop, and a 2998 PDA detector was used for analysis. Empower 3 for LC systems (Rev.B.03.01-SR1 (317)) was used for instrument control, data analysis, and data acquisition. Separation and quantitation were carried out on a Waters Symmetry C18 column, 100A, 5 µm, 4.6 mm × 250 mm at ambient temperature. A mixture of sol-A: 0.1% TFA in water, sol-B: IPA, and sol-C: 0.1% TFA in methanol in gradient elution mode (0–1 min sol-A: 50–50, sol-B: 20–20; 1–5 min sol-A: 50–0, sol-B: 20–90; 5–7 min sol-A: 0–0, sol-B: 90–90; 7–8 min sol-A: 0–50, sol-B: 90–20; 8–12 min sol-A: 50–50, sol-B: 20–20) was used. The flow rate was maintained at 0.6 mL min^−1^. The system was equilibrated and saturated with the mobile phase for 30 min before injection of the solutions. Quantification was achieved with PDA detection at 280 nm. Fifteen µL of the solutions were injected in duplicate. All samples were quantitated by the peak area based on interpolation from a five-point dilution series in ethanol of the calibration standard.

To quantitate INI-4001 adsorbed to A-SNP, unbound INI-4001 was determined using RP-HPLC after separating INI-4001/A-SNP via centrifugation. Briefly, 30 µL from each sample were transferred to a 0.5 mL microcentrifuge tube after brief vortexing to fully suspend the particles. The particles were then isolated by centrifugation at 960× *g* for 4 min, and 15 µL of the supernatant were transferred to a suitable HPLC vial and completed to 90 µL using 3% Triton X-100. The concentration of unbound INI-4001 was determined using the aforementioned HPLC method. To characterize antigen adsorption, the concentration of H7 in the supernatants was determined using a Thermo Scientific NanoDrop UV-Vis spectrophotometer at 280 nm. The percentage of H7 adsorbed was estimated relative to the H7 in the control samples.

### 2.5. Determination of INI-4001 Release Kinetics from INI-4001/A-SNP Formulations in Plasma

#### 2.5.1. Development and Optimization of a Bioanalytical Method for Quantitation of INI-4001 in Biological Samples

Serum was isolated from human blood obtained from healthy adult donors through a University of Montana Institutional Review Board (IRB)-approved protocol. Frozen human serum was thawed at room temperature and vortexed briefly prior to use. Calibration standards of INI-4001 ranging from 0.05 to 10 µg/mL were prepared by spiking 99 µL of human plasma with 1 µL of INI-4001 solution in 100% methanol. The spiked sample was vortexed for 1 min and incubated at 37 °C for 1 h. Acetone (600 µL) was added to the spiked serum sample and vortexed for 2 min to precipitate the serum proteins. The sample was centrifuged at 19,400× *g* (5417C, Eppendorf) for 20 min at room temperature, and the supernatant was collected and evaporated under vacuum using a Savant SpeedVac vacuum concentrator (Thermo Fisher Scientific) at ambient temperature. The dried residue was reconstituted and vortexed with 100 μL of methanol, and the amount of INI-4001 was quantified using a Waters Acquity Arc UHPLC system equipped with a Waters CORTECS C18 3.0 × 50 mm 2.7 µm column at 40 °C. This method used a gradient mobile phase using solvent A (0.5% 1 M ammonium formate and 9% methanol in 90.5% water) and solvent B (0.5% 1 M ammonium formate and 99.5% methanol). Elution was obtained by using the following gradient steps of solvents A and B: 85:15 (A:B) for the initial 2 min, 100% B for the next 10 min, and 85:15 (A:B) for the final 3 min at a flow rate of 1 mL/min. A detection wavelength of 280 nm was used for the study. The extraction efficiency of the spiked serum sample with INI-4001 was compared against an INI-4001 standard of a similar concentration in methanol. A squarate analogue of INI-4001 (UM-2014) was used as an internal standard.

#### 2.5.2. In Vitro Release Studies of INI-4001 from 50 nm Low- and High-Coating-Density INI-4001/A-SNP Formulations

In vitro release studies of INI-4001 from low and high coating density INI-4001/A-SNP formulations were performed using human plasma as the release medium to simulate in vivo conditions. For the in vitro release experiment, 100 µL of the indicated formulations were mixed with 1500 µL of human plasma in a closed glass vial at 37 °C and gently mixed at 30 rotations per minute. At different time points (0.25, 0.5, 1, 2, 4, 8, 12, 24, 48, 72, and 168 h), 120 µL of the release medium were collected and centrifuged at 19,400× *g* for 5 min to pellet the A-SNP (each time point had separate samples). The supernatant (100 µL), which contained released INI-4001, was collected and stored at −20 °C until further analysis by RP-HPLC. The amount of INI-4001 present in the samples was quantified by RP-HPLC after extraction using the protocol mentioned above.

#### 2.5.3. Mathematical Modeling of the Desorption Profile of INI-4001

To understand the release kinetics and to identify the mechanism of release of INI-4001 from the INI-4001/A-SNP formulations, the release profiles were fitted with various kinetic models, including zero order, first order, Higuchi, Korsmeyer–Peppas, and Hixson–Crowell, using a DD solver and a Microsoft Excel plugin [34]. The model with the highest regression coefficient (R^2^) value was considered to be the best fitting model. The n value of the Korsmeyer–Peppas model was calculated to study the release mechanism.

### 2.6. Isolation of Human PBMCs and Cytokine Analysis

Peripheral blood samples were collected from healthy adult donors. The samples were collected after approval by the University of Montana Institutional Review Board, and signed written informed consent was obtained from each donor. PBMCs were isolated from peripheral blood using Ficoll–Paque, as previously reported [35]. Cells were cultured in 96-well plates at a density of 5 × 10^5^ cells/well in RP-10. The indicated formulations were serially diluted and added to plated PBMCs, which were then incubated at 37 °C, 5% CO_2_ for 18–24 h. Cell culture supernatants were collected and stored at −20 °C. Secreted cytokine levels in the supernatants were measured using either a DuoSet TNF-α ELISA (R&D Systems, Minneapolis, MN, USA) or a VeriKine-HS Human IFN-α All-Subtype TCM ELISA Kit (PBL Assay Science) per the manufacturer’s instructions. ELISAs were read on a plate reader at 450 nm, and the cytokine concentration was calculated by fitting the standard curve OD values to a 4-parameter logistical model using the curve fitting software (XLfit, IDBS, Alameda, CA, USA).

### 2.7. Isolation of Murine BMDCs and Cytokine Analysis

C57BL/6 mice were purchased from the Jackson Laboratory under University San Diego Institutional Animal Care and Use Committees regulation. Murine BMDCs from C57BL/6 mice were prepared as described previously [36]. mBMDCs were plated in a 96-well plate with 5 × 10^5^ cells/200 µL/well. The indicated formulations were serially diluted starting from a concentration of 10 µM and added to the plated mBMDCs. LPS (10 µg/mL, LPS-EB, Invivogen) and 1V270 (TLR7 ligand) were used for controls. After incubating for 18 h at 37 °C under 5% CO_2_, the supernatants were collected and the levels of cytokines (IL-6, IL-12, TNF-α, and IFN-β) were determined by ELISA (Table S1).

### 2.8. In Vivo Vaccine Studies

Male and female 6–8-week-old BALB/c mice from Jackson Laboratory were used for all in vivo studies. Mice were housed in an AAALAC-accredited facility, and all procedures were performed in accordance with the University of Montana IACUC approved animal use protocol. Mice were immunized 3 times 14 days or 28 days apart (the standard or extended vaccination schedule is noted for each study) by intramuscular injection in the hind limb with 1 μg of H7 and 10 µg of INI-4001/A-SNP formulations. Blood was collected at 14 or 28 days post-injection and centrifuged at 10,000× *g* in Microtainer Serum Separator Tubes (BD Biosciences) to obtain serum, which was stored at −20 °C. At the noted harvest time, the mice were euthanized and the spleens and draining lymph nodes (dLNs) (inguinal and popliteal LNs on the injection side) were collected.

#### 2.8.1. H7-Specific Antibody ELISAs

Serum levels of H7-specific IgG, IgG1, and IgG2a antibody concentration were measured as follows. ELISA plates were prepared by coating with 100 µL of H7 at 1 µg/mL, washing in 0.05% Tween-20 in PBS, and blocking with SuperBlock (Scytek Laboratories). Plates were then incubated with diluted serum for 1 h followed by binding with anti-mouse IgG, IgG1, or IgG2a-HRP secondary antibody and detection using TMB Substrate (BD Biosciences). Absorbance was measured at 450 nm using a Molecular Devices SpectraMax 190 microplate reader, and antibody titers were determined by calculating the titer of each sample at OD 0.3 [7].

#### 2.8.2. Splenocyte and dLNs Cell Restimulation and Cytokine Analysis

The spleens were harvested from the vaccinated mice 5 or 21 days after tertiary injections (dp3) for the short and extended schedules, respectively. Single-cell splenocyte suspensions were prepared by mechanical digestion through a 100 µm cell strainer. Red blood cells were lysed by incubation with red blood cell lysis buffer (Sigma) for 5 min followed by washing in PBS. Cells were plated in a 96-well plate at 5 × 10^6^ cells/well in 200 µL RP-10. IFN-γ, IL-17, and IL-5 cytokines secreted by splenocytes cultured with 1 µg/mL H7 antigen were measured in the supernatant after 72 h of stimulation by MesoScale Discovery (MSD) U-PLEX Assay Platform.

### 2.9. Statistical Analysis

The statistics were analyzed using GraphPad Prism 8 software (San Diego, CA, USA). The results were confirmed for normality using the D’Agostino–Pearson omnibus normality test. Student’s *t*-test was used to compare 2 conditions, and one-way ANOVA was used to determine variances in means between more than 2 formulation groups (*p* < 0.05 *, < 0.01 **, < 0.001 ***, and < 0.0001 ****).

## 3. Results and Discussion

Despite their potential, the clinical translation of TLR7/8 agonists has been limited due to their high potency and lack of pharmacokinetic control, which together can result in systemic immunotoxic side effects from unregulated systemic cytokine release [13]. Bhagchandani et al. reviewed TLR7/8 agonist formulations that have advanced to the clinic as well as recent strategies aiming at enhancing their safety and efficacy using bioconjugates and nanoparticle formulations [14]. The lipid tail of the imidazoquinoline 3M-052 TLR7/8 agonist, for example, allows gradual delivery when formulated in oil-in-water emulsions or incorporated into the lipid bilayer of liposomes to control its distribution, preventing the systemic release of TNF-α and the detection of pro-inflammatory cytokines in the spleen [37]. In another example, Huang et al. showed that the immune potency of small-molecule TLR7 ligands was amplified when conjugated to silica nanoshells while the ligand-receptor specificity was retained, thereby broadening the potential agonistic application of these agents [15]. That amplification was both particle size dependent and ligand density dependent.

A reliable way to co-deliver antigens and adjuvants is to co-load them onto nanoparticles, which are increasingly used with innate immune stimulators to mitigate side effects and enhance vaccine efficacy. Herein, we describe a novel strategy for antigen and adjuvant co-delivery using a lipidated TLR7/8 agonist, INI-4001 (Figure 1A), that can be physically adsorbed and presented on the surface of tunable and biodegradable A-SNP without the need for direct chemical conjugation [22,38]. This silica-based vaccine delivery platform allows for the control of the TLR7/8 ligand and antigen valency while also achieving prolonged co-delivery of the adjuvant and antigen to the same APCs. We hypothesized that the lipidated nature of INI-4001 and the slow-release profile when co-adsorbed with the H7 antigen onto A-SNP would localize TLR7/8 presentation to the dLNs. This localization can promote antigen presentation, leading to improved efficacy and safety profiles of TLR7/8 adjuvanted subunit vaccines.

### 3.1. Preparation and Characterization of INI-4001/A-SNP Formulations

In our initial studies, different-sized A-SNP (50, 100, 200, and 1500 nm) were evaluated for their ability to achieve acceptable formulation characteristics with INI-4001-adsorbed A-SNP. In these studies, the concentration of INI-4001 and A-SNP remained fixed at a targeted concentration of 200 µM and 4 mg/mL, respectively. The adjuvant-coating density, however, varied with the different A-SNP sizes (Table S2). These INI-4001/A-SNP formulations were tested in hPBMCs to evaluate the adjuvant potency. The immune response observed with the 100 nm INI-4001/A-SNP formulation did not exhibit a clear distinction from its counterparts with sizes of 50 nm or 200 nm (Figure S1). Additionally, the 1500 nm INI-4001/A-SNP formulation displayed the least colloidal stability with quick flocculation, attributed to its larger particle size. Based on these initial formulation screening results, it was decided to select two A-SNP sizes (50 and 200 nm), which demonstrated acceptable formulation characteristics and distinct in vitro activity for the adsorbed INI-4001. The selected formulations were used to study the effect of particle size and adjuvant-coating density on the immunogenicity of INI-4001/A-SNP formulations. A-SNP were prepared as previously described [39], where bare SNP of each size were modified with APTES to functionalize their surface with cationic amine groups and facilitate the electrostatic adsorption of INI-4001 and H7 (both are anionic). Post-functionalization TEM images showed that the SNP size remained similar (Figure 1B). However, DLS hydrodynamic size measurements showed much larger particles that might have been caused by the presence of some flocculation that affected the reliability of these DLS measurements (Table 1). The successful surface functionalization with APTES was confirmed by measuring the zeta potential of the modified particles (Table 1). Furthermore, amine grafting was determined using a ninhydrin assay [39] and was estimated to be 0.386 and 0.455 mmol/g for the 50 and 200 nm A-SNP, respectively.

INI-4001, an anionic phospholipidated oxoadenine derivative, was adsorbed to A-SNP using a thin-film rehydration procedure followed by adsorption of the antigen using gentle mixing conditions to maintain its conformational stability. Two percent glycerol was used as the rehydration diluent to adjust the osmolality of the formulations to about 290 mOsmol/kg for parenteral administration. Choline bicarbonate was added to help with the ionization of INI-4001 and its adsorption to the cationic A-SNP. Thirty min of sonication at room temperature were found to be sufficient for INI-4001 adsorption onto A-SNP while avoiding heat-induced degradation of the adjuvant. After resuspension of the coated particles, the antigen H7 was co-adsorbed to INI-4001/A-SNP using end-over-end mixing at room temperature for 2 h. The adsorption of the antigen and adjuvant was evaluated after pelleting the particles by analyzing the free, un-adsorbed INI-4001 and H7 using RP-HPLC and UV-Vis spectroscopy, respectively. INI-4001 adsorbed efficiently (over 95%) to 50 and 200 nm INI-4001/A-SNP low and high density formulations, respectively. The adjuvant coating density determined the overall charge of the INI-4001/A-SNP formulations. The successful adsorption of anionic INI-4001 (−62.2 ± 6.8 mV) at low and high coating densities onto the cationic A-SNP was strongly indicated by changes in the zeta potential. A negative zeta potential was observed for the highly coated formulations, whereas the less coated formulations maintained the cationic charge of the A-SNP (Table 1). However, the adjuvant coating density did not appear to affect the degree of its adsorption to the A-SNP since comparable adsorption was achieved in both cases (Table 2). For example, the formulation of INI-4001/A-SNP-50 low, with a positive zeta potential of 26.6 ± 4.8 (Table 1), demonstrated adjuvant and antigen % adsorption of 95.4 ± 1.7 and 94.1 ± 7.6, respectively. However, the formulation of INI-4001/A-SNP-50 high, with an a negative zeta potential of −20.1 ± 6.2 (Table 1), showed a comparable adjuvant and antigen % adsorption of 101.4 ± 1.9 and 96.7 ± 4.4, respectively. Although we suspect that hydrogen bonding or hydrophobic interactions play a role in the adhesion of INI-4001 to A-SNP, we hypothesized that ionic interactions are critical for the rapid and efficient coating of the A-SNP surface. H7 is an anionic trimeric glycoprotein with a molecular weight of 195 kDa [40] and an isoelectric point (IEP) of 4.5 [41]. These physicochemical properties were expected to facilitate its adsorption onto the cationic A-SNP. As predicted, H7 at a 20 µg/mL concentration adsorbed efficiently (over 94%) onto the A-SNP formulations regardless of whether they were coated with INI-4001. The adjuvant-coating density and the apparent charge of the particles did not affect the degree of adsorption of the antigen, which suggests that mechanisms other than electrostatic interaction might be involved in achieving efficient adsorption of INI-4001 and H7 (Table 2).

### 3.2. INI-4001 Shows Slow-Release Kinetics in Plasma When Adsorbed onto A-SNP

Extraction of INI-4001 from plasma was performed by protein precipitation from an organic solvent. Initially, various organic solvents, including acetone, tetrahydrofuran, methanol, acetonitrile, and methyl *t*-butyl ether, were tested, and the observed extraction efficiency of INI-4001 from plasma was 86.9, 64.4, 23.0, 9.1, and 3.4%, respectively. Hence, acetone was selected as the organic solvent to extract INI-4001 from biological samples.

**Figure 2:**
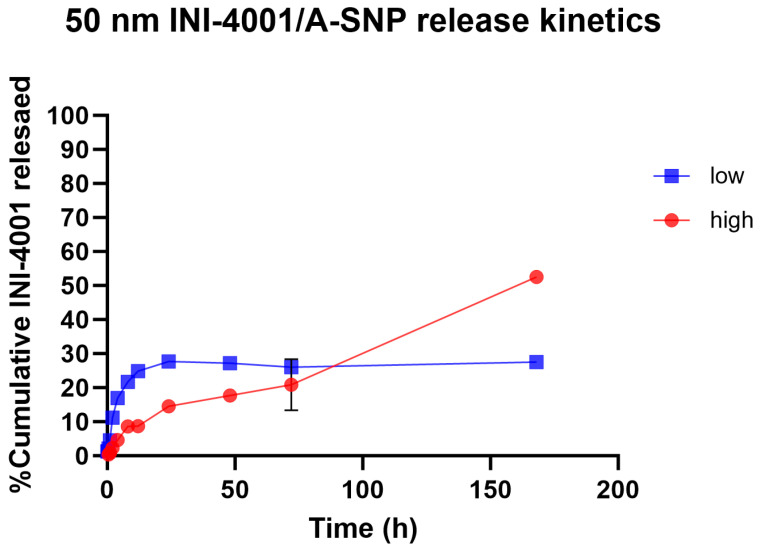
In vitro release profile in plasma of INI-4001 from 50 nm low and high coating density INI-4001/A-SNP formulations. Cumulative INI-4001 release is given as the mean ± SD (*n* = 2 independent replicates).

Slower and sustained-release profiles of adsorbed small- and large-molecule active pharmaceutical ingredients, including antigens, from A-SNP have been well documented [42,43,44]. The surface chemistry and charge of the two A-SNP sizes were comparable, with no significant differences in INI-4001 adsorption (as shown in Table 1 and Table 2). Next, we measured the release kinetics of the INI-4001/A-SNP formulations using 50 nm A-SNP. The in vitro release profiles of INI-4001 from the 50 nm INI-4001/A-SNP formulations are shown in Figure 2. The release profile suggests a difference in the nature of release patterns between the low and high coating density INI-4001/A-SNP. The release profile of INI-4001 from the high coating density formulation was sustained and lacked an initial burst release. At the end of the release kinetic study, 168 h, ~50% of INI-4001 was released. In contrast, the release profile of the low coating density formulation indicated a biphasic release. In the initial 8 h, a burst release of 20% was observed, followed by a sustained-release profile where only ~30% of INI-4001 was released after 168 h.

To have a better understanding of the mechanism of release of INI-4001 from the low and high coating density INI-4001/A-SNP formulations, the release profiles were fitted with various kinetic models, and the parameters are presented in Table 3. The release profile of INI-4001 from both the high and low coating density INI-4001/A-SNP showed a good fit to the Korsmeyer–Peppas model based on the high R^2^ values. The *n* value of 0.738 for the high coating density INI-4001/A-SNP indicates that the release of INI-4001 followed a non-Fickian transport, whereas the n value of 0.260 for the low coating density INI-4001/A-SNP suggests a Fickian diffusion, i.e., the release of INI-4001 in plasma was from a region of higher concentration to lower concentration [45].

### 3.3. INI-4001-Coating Density and A-SNP Size Affect IFN-α and TNF-α Induction in Human PBMCs

Previous studies demonstrated that the immunostimulatory properties of TLR7/8 ligands are influenced by the size and charge of drug carriers as well as the adjuvant coating density [15,46]. Such differences in biological activity and functional outcome can be attributed to various mechanisms, such as particle internalization or intracellular distribution in APCs that can impact signaling [15,47]. Therefore, we hypothesized that the size of SNP and the adjuvant coating density may influence the immune potencies of the TLR7/8 ligand INI-4001. To test this hypothesis, 50 and 200 nm A-SNP were coated with INI-4001 and human PBMCs were treated with aqueous INI-4001 control, blank A-SNP, or INI-4001/A-SNP of these various sizes. To determine the influence of the adjuvant-coating density, two different coating densities were tested for the 50 and 200 nm INI-4001/A-SNP: 0.02 and 0.2 nmol/cm^2^, defined as low and high coating density, respectively.

**Figure 3:**
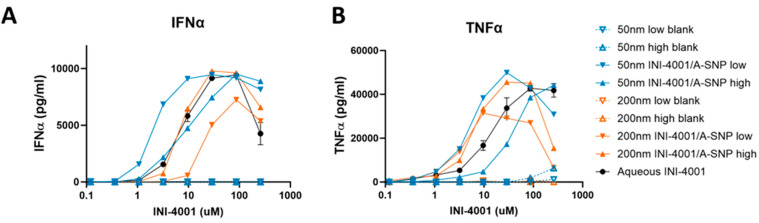
Effect of INI-4001-coating density and A-SNP size on IFN-α (**A**) and TNF-α (**B**) induction in hPBMCs. Freshly isolated hPBMCs were stimulated for 24 h with A-SNP coated with low or high density INI-4001/A-SNP (50 and 200 nm) formulations. TNF-α and IFN-α were quantified by ELISA from cell culture supernatants. Data shown is from a representative donor of 3.

Testing the adjuvanticity of INI-4001/A-SNP as a function of particle size or ligand-coating density revealed that some of these formulation conditions induced higher production of cytokines (IFN-α and TNF-α) compared to the INI-4001 aqueous control (Figure 3). A correlation was observed between ligand density and in vitro immune response that was also dependent on the size of the particles. The enhancement in IFN-α production and potency was more profound in the case of the 50 nm low density INI-4001/A-SNP formulation over the INI-4001 aqueous formulation with an EC-50 of 3.2 ± 0.7 and 34.0 ± 10.3, respectively (*p* < 0.05, Figure 3A and Table 4), whereas the 200 nm low density INI-4001/A-SNP formulation yielded the weakest IFN-α induction amongst the tested formulations (Figure 3A). TNF-α induction was higher for several INI-4001/A-SNP formulations than the aqueous control. However, it is noteworthy that the low density INI-4001/A-SNP for both sizes exhibited significantly higher TNF-α induction than the high density formulations (Figure 3B and Table 4).

These divergent results of the tested formulations, especially in the case of the 200 nm low-density INI-4001/A-SNP, in terms of IFN-α (a primary readout for TLR7 activation) and TNF-α (a primary readout for TLR8 activation) induction could have been due to differential uptake by specific APC types and, consequently, the targeting of TLR7 versus TLR8 endosomal receptors. TLR7 is mainly expressed in plasmacytoid DCs (pDCs) and B-cells, whereas TLR8 is primarily expressed in myeloid cells, such as monocytes, macrophages, and myeloid DCs [48]. Distinct physicochemical properties of the tested formulations, including size, charge, and adjuvant coating density, could have influenced uptake and targeting to these specific immune cells. Of note, the blank A-SNP without INI-4001 did not induce TNF-α or INF-α on their own, which makes them a suitable vaccine delivery system with limited interference with the activity of the TLR7/8 agonist (Figure 3A,B). Surface modifications of SNP, including decorating the surface of amorphous SNP with functional groups such as amine, can mitigate any of their toxic impactions [49]. The effect of these formulation parameters on cell viability was evaluated, and it was found that stimulation of hPBMCs with A-SNP formulations had no significant effect on hPBMC viability (Figure S2).

### 3.4. Evaluation of INI-4001/A-SNP Formulations in mBMDCs

**Figure 4:**
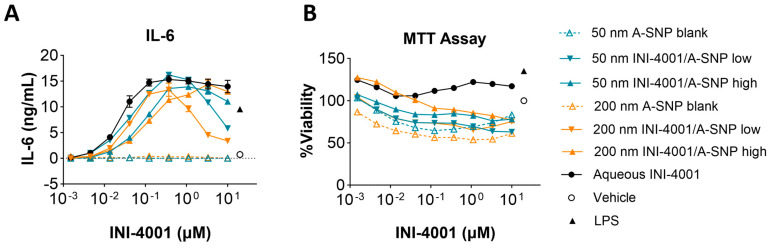
Cytokine release by 50 and 200 nm INI-4001/A-SNP formulations from mBMDCs. mBMDCs (5 × 10^5^/200 µL/well) were incubated with 50 and 200 nm INI-4001/A-SNP formulations overnight, and TNF-α, IL-6, and IL-12 release was determined by ELISA. A-SNP only, INI-4001 aqueous formulation in the same vehicle, and LPS served as controls. (**A**) Representative data of IL-6 releases by mBMDCs treated with 50 and 200 nm INI-4001/A-SNP. (**B**) MTT assay for different formulations.

Two different A-SNP sizes, 50 and 200 nm with adsorbed INI-4001, were tested in mBMDCs before evaluating them in vivo to confirm analogous activity between mouse and human cells. INI-4001 was adsorbed on the surface of 50 and 200 nm A-SNP at low or high density, as described in the previous sections. mBMDCs were incubated with serially diluted INI-4001/A-SNP formulations. The cells were incubated overnight, and TNF-α, IL-6, IL-12, and IFN-β levels in the culture supernatant were measured by ELISA. Low density INI-4001 on 50 nm and 200 nm A-SNP showed significantly higher IL-6 production than the high density formulations, with a comparable level of cytokine induction to that of INI-4001 aqueous control (Figure 4A). In these experiments, the size of the A-SNP did not influence the potency of the INI-4001/A-SNP formulations. INI-4001 on 50 and 200 nm A-SNP showed slightly lowered cell viability at a higher concentration by MTT assay, which might correlate with the decreased cytokine induction at the higher concentration (Figure 4B). TNF-α and IL-12 release exhibited similar trends to IL-6 (Table 5). The IFN-β levels were below the detection limit. Of note, TLR8 is mostly inactive in mice and TLR7 is widely expressed on monocytes, macrophages, and mDCs [50]. Therefore, the differential cell targeting from hPBMCs noted above (IFN-α vs. TNF-α) would not be expected in mBMDCs. Indeed, the 50 and 200 nm formulations with INI-4001 exhibited similar cytokine release profiles in mBMDCs. In addition, since TLR8 is mostly inactive in murine cells [50], cytokine release by INI-4001/A-SNP was below the detection limits in TLR7-deficient mBMDCs, indicating that the cytokine release of these formulations was TLR7 dependent (Figure S3).

Overall, these cumulative results demonstrate that the use of A-SNP is likely to enhance the response to INI-4001, and factors such as particle size and proper adjuvant-coating density should be carefully optimized. In this work, the low adjuvant coating density with the small-size 50 nm A-SNP in particular seemed to perform best overall, especially in hPBMCs, which could be attributed to better uptake by immune cells. However, the in vivo context is more complicated, involving potential differences in trafficking and perhaps even in the subsets of cells that are likely to interact with the particles. Therefore, further studies are imperative to gain a comprehensive understanding of the differences in immune responses induced by these varying formulation conditions.

### 3.5. INI-4001/A-SNP Formulations Enhance Humoral and Cellular Immune Responses

The above data demonstrate that the adsorption of TLR7/8 ligand onto A-SNP can enhance the in vitro potency of innate immune stimulatory activity in human and murine immune cells. Furthermore, A-SNP can support prolonged local depot effects that may enhance immune-stimulatory effects in vivo [51]. Therefore, an immunization model using the hemagglutinin influenza antigen H7 was performed to study the effects of INI-4001/A-SNP size and adjuvant coating density on the in vivo antigen-specific humoral and cellular immune response. IgG2a and IgG1 were used as indicators for Th1-type and Th2-type immune responses, respectively [52]. The Th1-type response, regulating cellular immunity, contributes to the development and activation of cytotoxic T cells [53]. Conversely, Th2-type immunity regulates the humoral immune response and induces the proliferation and differentiation of B cells [54].

These studies aimed to evaluate A-SNP as a co-delivery platform for TLR7/8-based adjuvants and the influenza antigen H7 and to identify the optimal INI-4001-coating density and A-SNP particle size. We initially screened the formulations using the prime-boost schedule with a 14-day interval (referred to as a 14-day regimen) and subsequently compared selected formulations using an extended schedule (28-day interval, referred to as a 28-day regimen), where the depot effects by INI-4001/A-SNP formulations on immune response could be better demonstrated. In both immunization studies, with 14-day and 28-day regimens, mice were immunized with 1 µg of influenza A H7 trimer adsorbed onto A-SNP (50 and 200 nm) with a low or high density of a dose of 10 nmol of INI-4001. The INI-4001 dose was selected based on previous dose escalation studies of its emulsion formulation [18].

#### 3.5.1. Humoral Responses Using 14-Day Regimen

**Figure 5:**
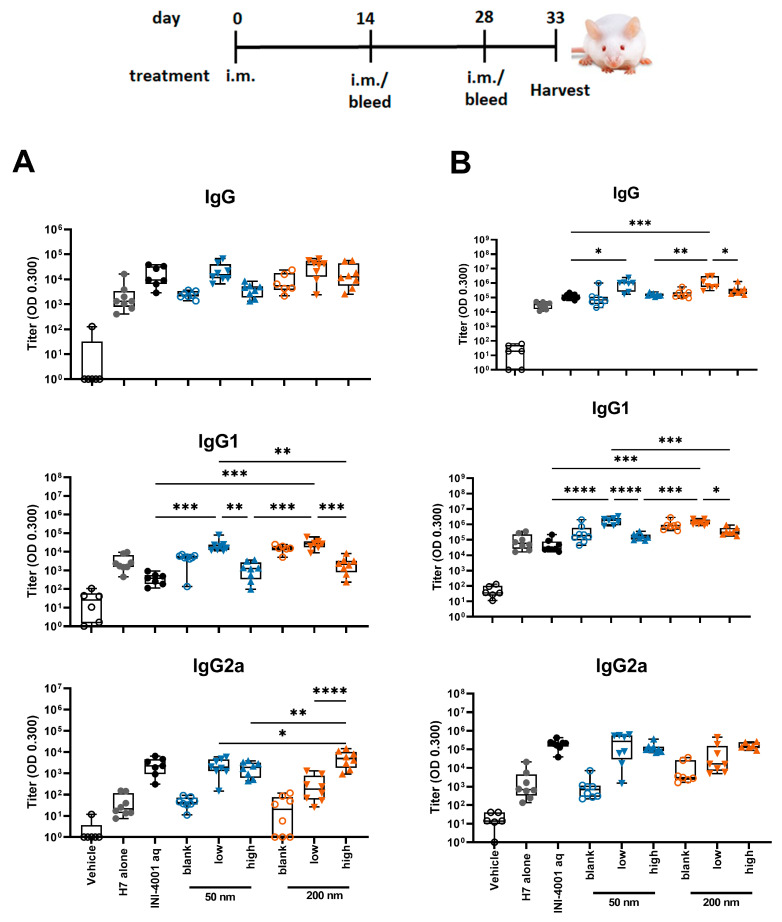
H7-specific antibody titers 14 days post-primary (**A**) or post-secondary (**B**) vaccination. A-SNP were formulated with a high or low coating density of 10 nmol of INI-4001. The mice were immunized with 1 µg of influenza/A H7 trimer adsorbed to INI-4001/A-SNP formulations at the described particle size and coating density. One-way ANOVA of log-transformed data followed by uncorrected Fisher’s multiple comparisons was used to calculate statistically significant differences between the INI-4001-containing groups (black) and the H7 antigen-only group (red). * *p* < 0.05; ** *p* < 0.01; *** *p* < 0.001; **** *p* < 0.0001. *n* = 6–8.

In the study using the 14-day interval schedule, the low density 50 and 200 nm INI-4001/A-SNP formulations produced markedly enhanced antigen-specific IgG and IgG1 antibody responses both post-primary and -secondary vaccinations in comparison to non-adjuvanted H7 antigen, high density INI-4001/A-SNP, and the aqueous INI-4001 groups (Figure 5). These results are consistent with the immunogenicity trends observed in in vitro studies. For IgG2a responses, INI-4001 in both aqueous and high-density 200 nm A-SNP formulations demonstrated significantly higher IgG2a titers 14 days post-primary vaccination than the non-adjuvanted H7 antigen group, whereas the INI-4001 aqueous and low-density 50 nm A-SNP formulations demonstrated significantly higher titers 14 days post-secondary vaccination (Figure 5).

#### 3.5.2. Humoral Responses Using 28-Day Regimen

**Figure 6:**
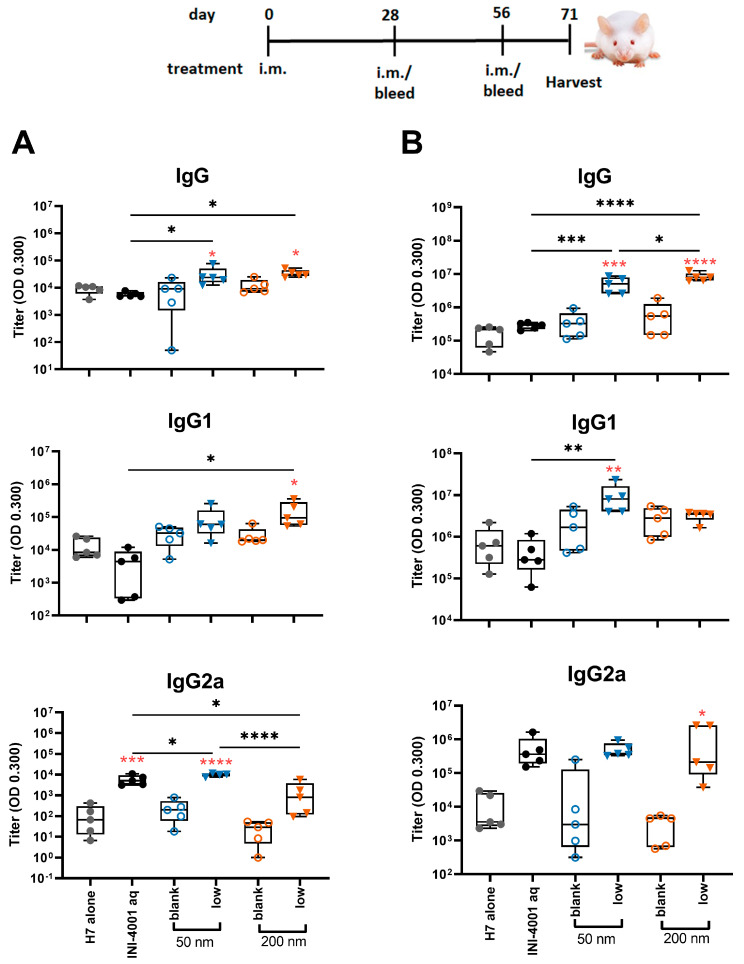
H7-specific antibody titers 28 days post-primary (**A**) or post-secondary (**B**) vaccination. The mice were immunized with 1 µg of influenza/A H7 trimer adsorbed onto INI-4001/SNP in the described particle size and coating density. One-way ANOVA of log-transformed data followed by uncorrected Fisher’s multiple comparisons was used to calculate statistically significant differences between the INI-4001 containing groups (black) and the H7 antigen-only group (red). * *p* < 0.05; ** *p* < 0.01; *** *p* < 0.001; **** *p* < 0.0001. *n* = 5.

In the study using the 28-day regimen, the 50 and 200 nm low-density INI-4001/A-SNP formulations were compared against H7 alone, blank A-SNP formulations without INI-4001, and the aqueous INI-4001 groups. The extended schedule consisted of three injections 28 days apart. At 28 days post primary injection, both the 50 and 200 nm INI-4001/A-SNP formulations induced similar levels of IgG and IgG1 serum antibodies that were significantly higher than those induced by the non-adjuvanted antigen- alone and aqueous INI-4001 groups (Figure 6A). Both the aqueous INI-4001 and 50 nm INI-4001/A-SNP formulations induced significantly higher IgG2a titers than the other groups (Figure 6A). Furthermore, at 28 days post-secondary injection, both 50 and 200 nm INI-4001/A-SNP formulations induced significantly higher levels of H7-specific IgG serum antibodies compared to the antigen- alone and aqueous INI-4001 groups, which were more pronounced for the larger size (17.2- and 27.2-fold, respectively; Figure 6B and Figure S4). Interestingly, lower IgG1 antibody titers were observed 28 days post-secondary injection in the case of the 200 nm INI-4001/A-SNP formulation relative to the 50 nm size (Figure 6B and Figure S4). For IgG2a responses observed 28 days post-secondary injection, only the 200 nm INI-4001/A-SNP formulation exhibited significantly higher IgG2a titers compared to the non-adjuvanted H7 antigen group (Figure 6B), indicating enhanced Th1 skewing. This switch toward a more Th1-biased response in IgG subclass might be attributable to the slower kinetics of humoral immune response stimulation facilitated by the larger size of the 200 nm INI-4001 formulation relative to the smaller size of the 50 nm counterpart.

Taken together, the results of the humoral responses indicate that the low coating-density 50 nm and 200 nm INI-4001/A-SNP formulations presented superior adjuvant efficacy among the tested formulations with a balanced Th1/Th2-type immunity, where the magnitude, type, and kinetics of the induced humoral immune response were highly dependent on the size of the A-SNP, the INI-4001-coating density, and the vaccination schedule used.

#### 3.5.3. Antigen-Specific Splenic T Cell Responses Using the 14-Day Regimen

**Figure 7:**
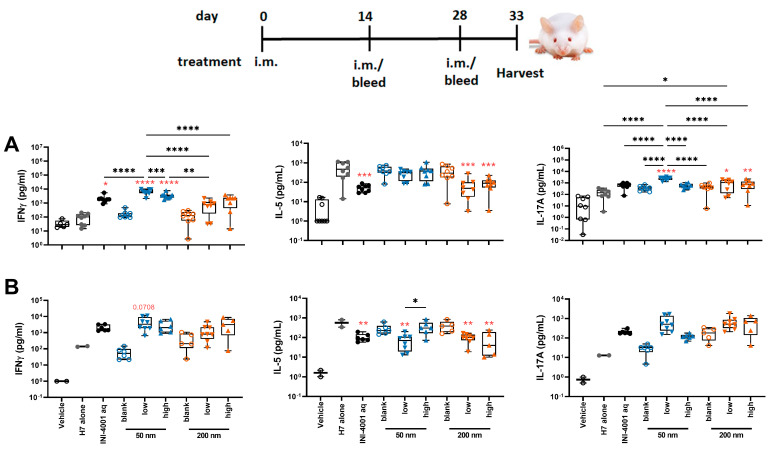
T cell cytokines produced by restimulated splenocytes (**A**) and dLNs (**B**) ex vivo (14-day regimen). Five days post-tertiary immunization, the spleens or dLNs were removed and processed. The splenocytes or dLNs were restimulated with H7 trimer for 72 h at 37 °C, 5% CO_2_. Cell culture supernatants were assessed for cytokine production by multiplex ELISA. One-way ANOVA of log-transformed data followed by uncorrected Fisher’s multiple comparisons was used to calculate statistically significant differences between the INI-4001-containing groups (black) and the H7 antigen-only group (red). * *p* < 0.05; ** *p* < 0.01; *** *p* < 0.001; **** *p* < 0.0001. *n* = 5–8.

To evaluate the cellular immunity of these different INI-4001/A-SNP formulations, IFN-γ, IL-17, and IL-5 production was measured in splenocytes and dLNs isolated from mice treated according to the 14-day vaccination schedule. The 50 nm low-density INI-4001/A-SNP formulation induced the most pronounced production of IFN-γ and IL-17 compared to the non-adjuvanted H7 antigen- alone and aqueous INI-4001 groups in both the spleens (Figure 7A) and the dLNs (Figure 7B), indicative of a strong Th1/Th17-biased immunity. As IL-5 is a Th2 cytokine, the IL-5 data showed opposite trends to IFN-γ and IL-17, with no statistical difference between the INI-4001/A-SNP formulations and aqueous INI-4001 (Figure 7A,B).

Taken together, the results of the T cell responses indicate the induction of a strong Th1/Th17-type immunity for the low density 50 nm INI-4001/A-SNP formulation over the other groups. Based on this perspective, further in-depth mechanism-of-action studies focused on understanding the impact of these different formulations on broadening and redirecting adaptive immune responses may represent a key aspect in advancing these adjuvanted vaccine formulations. In fact, adjuvant formulations can be tailored to enhance the required immune response for individual causative infectious agents.

## 4. Conclusions

New vaccine delivery technologies that can induce broader protection against serious infectious diseases such as influenza are urgently needed. Stimulation of TLRs is one successful strategy for initiating and directing immune responses that are essential for vaccine adjuvants. The present report has shown that the adjuvant immunogenicity of our novel lipidated TLR7/8 ligand, INI-4001, can be amplified when adsorbed and presented on tunable and biodegradable A-SNP. Two A-SNP sizes (50 and 200 nm) coated with INI-4001 at low and high densities and two vaccination regimens (14 and 28 days) were investigated to gain insight about the influence of these aspects on the adjuvanticity of the INI-4001/A-SNP formulations when paired and co-delivered with the influenza antigen H7. A-SNP were able to achieve efficient co-adsorption of INI-4001 and the influenza antigen H7 with a slow in vitro release kinetics profile in plasma. The adjuvant potency was both particle size dependent and ligand density dependent, where low-density 50 nm INI-4001/A-SNP showed the greatest overall agonistic activities in vitro, as indicated by the stronger induction of cytokines in hPBMCs and mBMDCs. In our in vivo immunization studies using the 14-day regimen, H7-specific antibody titers indicated that low-density 50 nm INI-4001/A-SNP formulations presented superior adjuvant efficacy among the tested formulations with a balanced Th1/Th2-type response. Furthermore, antigen-specific cellular immunity readouts suggested the induction of a strong Th1/Th17-type immunity for the low-density 50 nm INI-4001/A-SNP over the other groups. For the 28-day regimen, the low-density INI-4001/A-SNP formulations resulted in the highest IgG2a response post-primary and post-secondary vaccinations for the 50 and 200 nm sizes, respectively. These data together demonstrate that co-adsorption of TLR7/8 ligands and antigens on the surface of customizable inorganic A-SNP can induce enhanced humoral and cellular immune responses. This implies a broader immunogenicity as an adjuvanted subunit vaccine for influenza and other indications. In this fashion, factors such as particle size, adjuvant-coating density, and vaccination regimen can be tailored to control the induction of desirable antigen-specific immune responses. Additional validations regarding the efficacy of these adjuvanted vaccine formulations are currently in progress. This includes conducting neutralization assays and challenge studies in a suitable animal model. These studies are crucial for gaining insights into the mechanisms underlying the protection provided by these formulations.

## Figures and Tables

**Table 1:** Description and characterization results of the INI-4001/A-SNP formulations.

Formulation	A-SNP Conc. (mg/mL)	INI-4001 Conc. (µM)	INI-4001-Coating Density nmol/cm^2^	TEM Size (nm) ^a^	Z-Average Diameter (nm)	PDI	Zeta Potential (mV) ^b^	pH
A-SNP-50	10.0	---	---	48.9 ± 2.5	215	0.270	46.7 ± 5.4	5.9
A-SNP-200	40.0	---	---	199.6 ± 15.3	997	0.422	61.5 ± 4.9	6.1
INI-4001 aqueous control	NA	226.3	---	---	73	0.163	−62.2 ± 6.8	8.2
INI-4001/A-SNP-50 high	1.0	221.2	0.20	55.9 ± 2.9	246	0.256	−20.1 ± 6.2	6.2
INI-4001/A-SNP-50 low	10.0	259.8	0.02	48.3 ± 2.4	1107	0.334	26.6 ± 4.8	5.8
INI-4001/A-SNP-200 high	4.0	277.6	0.21	209.3 ± 10.0	631	0.249	−34.1 ± 5.4	6.0
INI-4001/A-SNP-200 low	40.0	242.6	0.02	209.8 ± 18.3	1305	0.608	47.0 ± 3.9	6.0

^a^ Means ± SEM (*n* = 21). ^b^ Means ± SEM of 3 measurements.

**Table 2:** Adsorption results of INI-4001 and H7 to different sizes A-SNP at low or high adjuvant-coating density. Two replicates (from two different sample sets) mean ± SEM.

Formulation	INI-4001 % Adsorption ^a^	H7 % Adsorption ^a^
A-SNP-50 blank	---	97.7 ± 3.1
A-SNP-200 blank	---	98.2 ± 2.4
INI-4001/A-SNP-50 high	101.4 ± 1.9	96.7 ± 4.4
INI-4001/A-SNP-50 low	95.4 ± 1.7	94.1 ± 7.6
INI-4001/A-SNP-200 high	101.1 ± 3.0	97.7 ± 2.0
INI-4001/A-SNP-200 low	100.7 ± 1.8	97.8 ± 3.2

^a^ Means ± SEM of 2 separate determinations.

**Table 3:** In vitro release parameters for 50 nm low and high coating density INI-4001/A-SNP formulations in human plasma.

Formulation	Zero-Order (R^2^)	First Order (R^2^)	Higuchi (R^2^)	Korsmeyer–Peppas	
				R^2^	*n*
INI-4001/A-SNP high	0.9520	0.9717	0.9299	0.9866	0.738
INI-4001/A-SNP low	0.5178	0.3111	0.4811	0.8325	0.260

**Table 4:** Effect of INI-4001-coating density and A-SNP size on IFN-α and TNF-α induction of EC-50 in hPBMCs. EC-50 (µM) is shown as mean ± SEM of 3 independent experiments. * Denotes *p* < 0.05 by Student’s *t* test versus INI-4001/A-SNP high.

	50 nm INI-4001/A-SNP		200 nm INI-4001/A-SNP		
Cytokine	Low	High	Low	High	Aqueous INI-4001
IFN-α	3.2 ± 0.7	40.2 ± 30.9	13.6 ± 7.6	15.0 ± 8.8	34.0 ± 10.3
TNF-α	4.6 ± 1.5 *	132.1 ± 67.2	1.6 ± 0.8 *	14.5 ± 5.6	21.4 ± 8.7

**Table 5:** EC50 of 50 and 200 nm INI-4001/A-SNP formulations from mBMDCs. A summary of 2–4 independent assays is presented. EC50 (µM) was calculated by Prism 8. * Denotes *p* < 0.05 by Student’s *t* test versus INI-4001/A-SNP high.

	50 nm INI-4001/A-SNP		200 nm INI-4001/A-SNP		
Cytokine	Low	High	Low	High	Aqueous INI-4001
IL-6	40 ± 10 *	133 ± 23	20 ± 2 *	72 ± 15	19 ± 3
TNF-α	49 ± 15 *	117 ± 13	35 ± 5	95 ± 25	48 ± 12
IL-12	62 ± 31 *	58 ± 20	22 ± 3	52 ± 27	<1

## Data Availability

The data presented in this study are available from the corresponding author upon request.

## Supplementary Materials

The following supporting information can be downloaded at: https://www.mdpi.com/article/10.3390/pharmaceutics16010107/s1, Table S1: Reagents used in ELISA; Table S2: Description and adjuvant-coating density of the initial INI-4001/A-SNP formulations; Figure S1: Cytokine response of human PBMCs exposed to INI-4001/A-SNP formulations of various sizes (50, 100, 200, and 1500 nm) for 24 h. Cell culture supernatants were analyzed by ELISA for the presence of IFNα (A) as a primary readout for TLR7 activation and TNFα (B) as a readout for TLR8 activation. Data are the average of two experiments from one representative donor; Figure S2: Effect of INI-4001/A-SNP formulations on the viability of human PBMCs. PBMCs were cultured with serially diluted SNP formulations for 24 h and cell viability was determined using the CellTiter Glo assay system. Viability is expressed as percent relative luminescent units (RLUs) compared to PBMCs incubated for 24 h without the tested samples. Data are shown as mean ± SD of 3 independent experiments. Color should be used in print; Figure S3. IL-12 release by 50 and 200 nm INI-4001/A-SNP in TLR7-deficient mBMDCs. Wild-type and TLR7-deficient mBMDCs were incubated with the formulations overnight, and IL-12 release was determined by ELISA. A/SNP alone, LPS, and 1V270 (TLR7 ligand) served as controls; Figure S4: (A) Ratio of H7-specific antibody titers of the 50 and 200 nm low-density INI-4001/A-SNP formulations over the INI-4001 aqueous control 14 vs. 28 days post-primary or post-secondary vaccination. A scatter plot of the mean values of the IgG2a titers (X-axis) and IgG1 (Y-axis) for each formulation post-primary (B) or post-secondary (C) immunization was generated to show the adjuvant potency distribution.
