# Supplementary material for: Co-Delivery of a Novel Lipidated TLR7/8 Agonist and Hemagglutinin-Based Influenza Antigen Using Silica Nanoparticles Promotes Enhanced Immune Responses
Source: Pharmaceutics. 2024 Jan 13;16(1):107. doi: 10.3390/pharmaceutics16010107 (PMC10819884; doi:10.3390/pharmaceutics16010107)
Supplement: Supplementary file 1 [file pharmaceutics-16-00107-s001.zip › pharmaceutics-2792635-supplementary.pdf]

Co-Delivery of a Novel Lipidated TLR7/8 Agonist and Hemagglutinin-based Influenza Antigen Using Silica Nanoparticles Promotes Enhanced Influenza-Specific Immune Responses

Walid M. Abdelwahab[1,2], Sarah Auclair[1,2], Timothy Borgogna[1,2], Karthik Siram[1,2], Alexander Riffey[1,2], Hélène G. Bazin[3], Howard B. Cottam[4], Tomoko Hayashi[4], Jay T. Evans[1,2,3], David J. Burkhart[1,2,3*]

[1]Center for Translational Medicine, [2]Department of Biomedical and Pharmaceutical Sciences, University of Montana, 32 Campus Drive, Missoula, MT 59802, [3]Inimmune Corporation, 1121 East Broadway, Missoula, MT 59812, [4]Moores Cancer Center, University of California San Diego, La Jolla, CA 92093-0809

Table S1. Reagents used in ELISA

Reagents	Dilution factor	Source	Catalog #
Capture antibodies			
Purified rat anti-mouse TNFα	125	Thermo Fisher Scientific	14-7325-85
Purified rat anti-mouse IL-12 p40/p70	200	BD Biosciences	551219
Purified rat anti-mouse IL-6	100	BD Biosciences	554400
Detecting antibodies			
Biotin mouse anti- mouse TNFα	250	Thermo Fisher Scientific	14-7325-86
Biotin rat anti-mouse IL-12 p40/p70	1000	BD Biosciences	554476
Biotin rat anti-mouse IL-6	1000	BD Biosciences	554402
Other reagents			
Streptavidin, HRP	1000	Thermo Fisher Scientific	43-4323
KPL SureBlue™ TMB Peroxidase Substrate		Seracare	5120-0077

Table S2. Description and adjuvant coating density of the initial INI-4001/A-SNP formulations

Formulation	A-SNP Conc. (mg/mL)	INI-4001 Conc. (µM)	INI-4001 coating density nmol/cm^2
50 nm INI-4001/A-SNP	4.0	234	0.05
100 nm INI-4001/A-SNP	4.0	203	0.09
200 nm INI-4001/A-SNP	4.0	232	0.21
1500 nm INI-4001/A-SNP	4.0	159	1.05

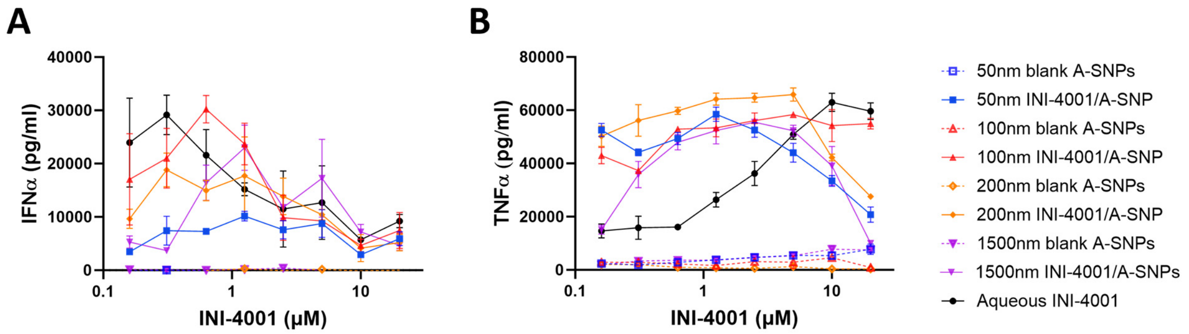

Figure S1. Cytokine response of human PBMCs exposed to INI-4001/A-SNP formulations of various sizes (50, 100, 200, and 1500 nm) for 24 h. Cell culture supernatants were analyzed by ELISA for the presence of IFNα (A) as a primary readout for TLR7 activation and TNFα (B) as readout for TLR8 activation. Data is the average of two experiments from one representative donor.

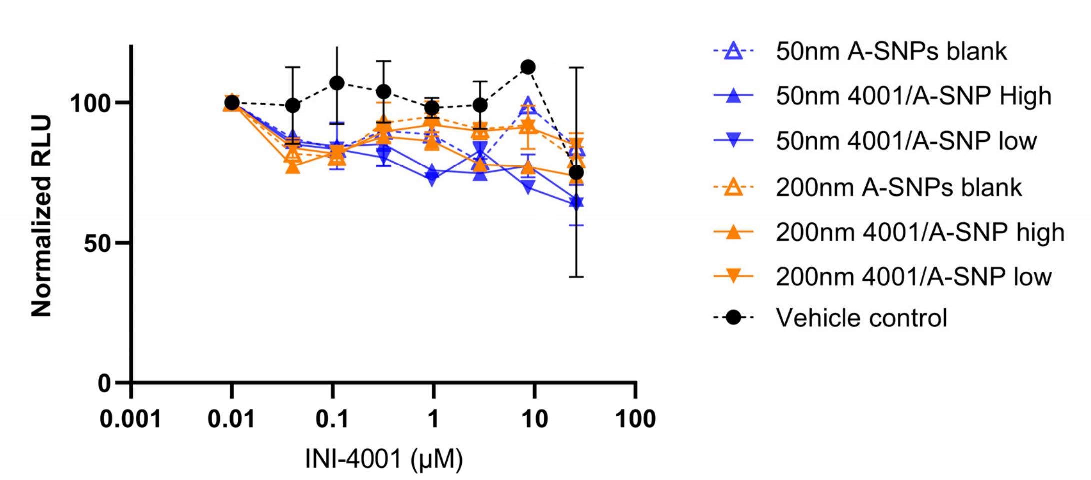

Figure S2. Effect of INI-4001/A-SNP formulations on viability of human PBMCs. PBMCs were cultured with serially diluted SNP formulations for 24 hours and cell viability determined using the CellTiter Glo assay system. Viability is expressed as percent relative luminescent units (RLU) compared to PBMCs incubated 24 hours without the formulations. Data shown as mean ± SD of 3 independent experiments. Color should be used in print.

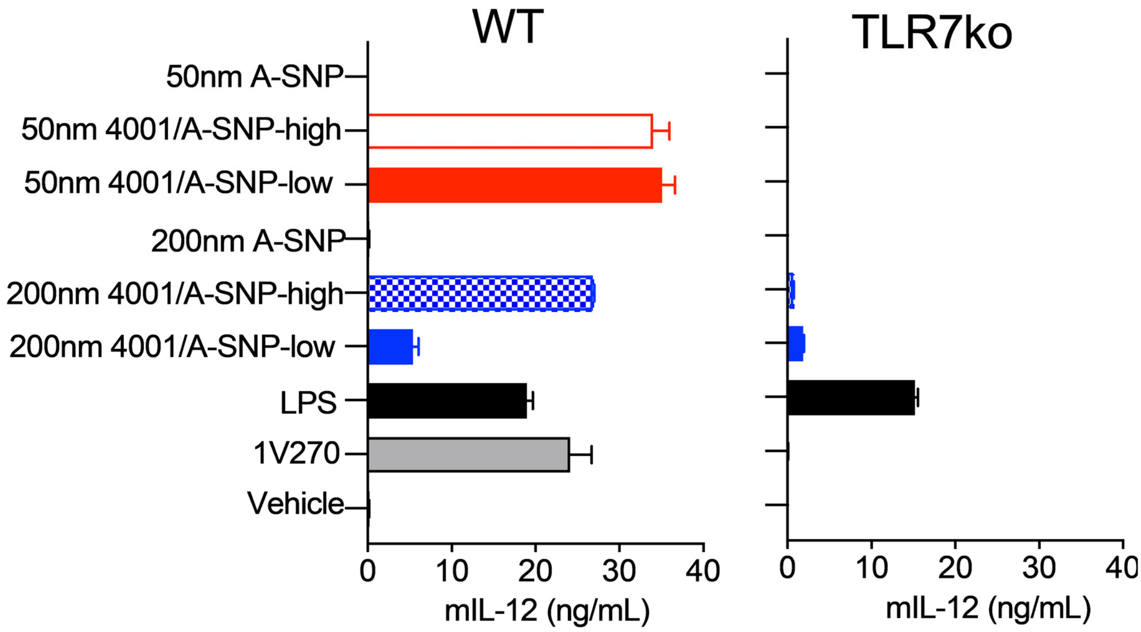

Figure S3. IL-12 release by 50, and 200nm INI-4001/A-SNP in TLR7 deficient mBMDC. Wild type and TLR7 deficient mBMDC were incubated with the formulations overnight, and IL-12 release was determined by ELISA. A/SNP alone, LPS, and 1V270 (TLR7 ligand) served as controls.

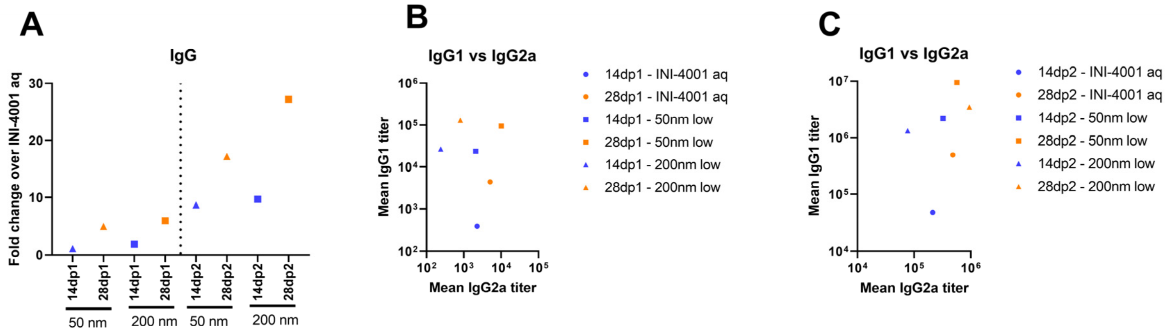

Figure S4: (A) Ratio of H7-specific antibody titers of the 50 and 200 nm low-density INI-4001/A-SNP formulations over the INI-4001 aqueous control 14 vs 28 days post primary or secondary vaccination. Scatter plot of the mean values of the IgG2a titers (X-axis) and IgG1 (Y-axis) for each formulation post-primary (B) or post-secondary (C) immunization was generated to show the adjuvant potency distribution.
